# Painful Os Peroneum Syndrome: Underdiagnosed Condition in the Lateral Midfoot Pain

**DOI:** 10.1155/2016/8739362

**Published:** 2016-07-05

**Authors:** Francisco Abaete Chagas-Neto, Barbara Nogueira Caracas de Souza, Marcello Henrique Nogueira-Barbosa

**Affiliations:** ^1^Division of Radiology, Antonio Prudente Hospital, Fortaleza, CE, Brazil; ^2^School of Medicine, Division of Radiology, University of Fortaleza, Fortaleza, CE, Brazil; ^3^School of Medicine, Division of Radiology, Christus University Center, Fortaleza, CE, Brazil; ^4^Division of Radiology, Internal Medicine Department, Ribeirao Preto Medical School, Sao Paulo University, Ribeirão Preto, SP, Brazil

## Abstract

Os peroneum is an accessory ossicle located within the peroneus longus tendon. The painful os peroneum syndrome (POPS) results from a wide spectrum of conditions, including fractures, diastases, and other causes. POPS can result in tenosynovitis or discontinuity of the peroneus longus tendon with a clinical presentation of pain in the lateral aspect of the midfoot. Authors report a typical case of POPS, illustrating this entity through different imaging methods (radiographs, ultrasound, and magnetic resonance imaging). We emphasize the prevalence of this ossicle and discuss painful complications.

## 1. Introduction

Os peroneum is an accessory ossicle located within the substance of the peroneus longus tendon. Os peroneum is identified in 4.7–30% of normal feet [1] and is bipartite in approximately 30% of cases and unilateral in 40%. Its fully ossified form is found in about 26% of population [2].

Painful os peroneum syndrome (POPS) results from a wide spectrum of conditions, including fractures or diastases, and may result in tenosynovitis or even rupture of the peroneal tendon [1].

This syndrome should be considered in patients with pain in the lateral aspect of the midfoot. A positive physical examination reveals pain during palpation of the ossicle; however, it is easily overlooked.

Imaging, such as radiographs, ultrasonography, and magnetic resonance imaging (MRI), plays an important role in the diagnosis and in the other associated conditions.

This report aims to illustrate, using different imaging methods, a typical case of POPS, to raise the degree of suspicion of this entity and highlight possible related complications.

## 2. Case Report

**Figure 1:**
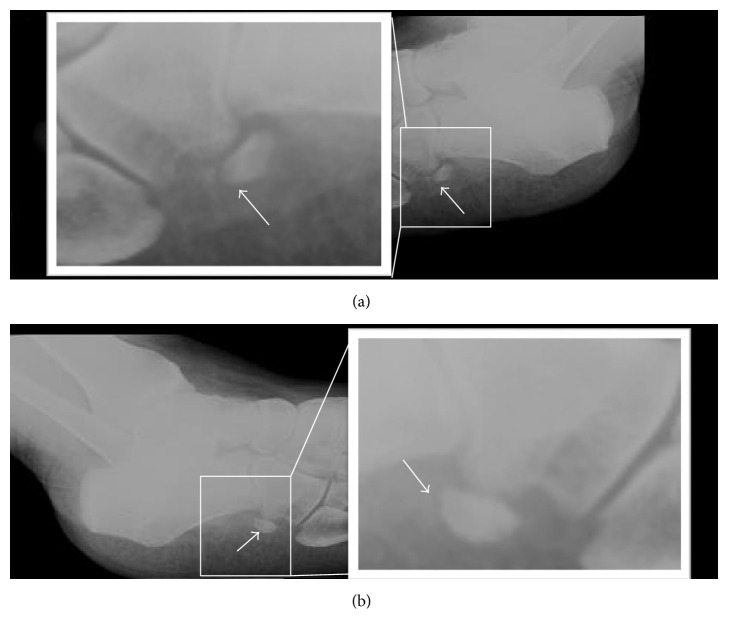
60-year-old female plain film of the feet in an oblique view. (a) Right foot: complaint side, showing an irregular and fragmented os peroneum with heterogeneous density (arrow). (b) Left foot: comparative contralateral side, showing a regular and complete os peroneum with regular contours and homogeneous density (arrow).

A 60-year-old female patient presented with progressive pain in the lateral aspect of the right midfoot. She denied any history of recent trauma, sprain, or high-impact sport activity. She was evaluated by an orthopedist who requested plain films of the right foot (Figure 1(a)). The plain film showed the presence of an accessory ossicle in the lateral aspect of the midfoot, located in the path of the peroneal tendons with cortical discontinuity, fragmentation, irregular margins, and heterogeneous density. Simple contralateral comparative radiograph of the left foot also showed the same accessory bone; however, there was intact margins and homogeneous density (Figure 1(b)).

**Figure 2:**
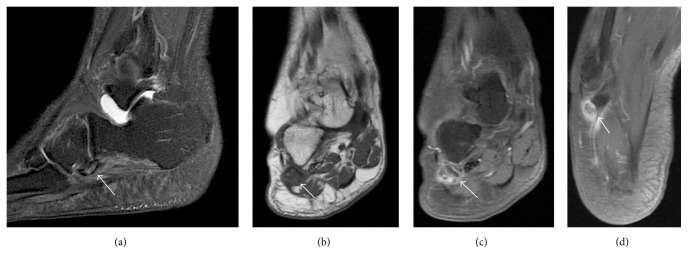
60-year-old female right midfoot MRI. (a) Sagittal T2; (b) coronal T1; (c) fat suppressed T1 postgadolinium; and (d) axial fat suppressed T1 postgadolinium. Arrows show the os peroneum within the peroneal tendon, with irregular contours, bone marrow edema, and intense enhancement of the adjacent soft tissues, characterizing inflammatory changes. The peroneus longus tendon is thickened and heterogeneous, consistent with associated tendinopathy.

Following the plain film, an MRI was performed for soft tissue evaluation. The accessory ossicle was identified within the peroneus longus tendon in the lateral aspect of the midfoot. It showed diffuse marrow edema, irregular margins, and cortical discontinuity. Also, there was edema and intense enhancement in the adjacent soft tissues (Figure 2). The peroneus longus tendon was thickened and heterogeneous, consistent with tendinopathy.

**Figure 3:**
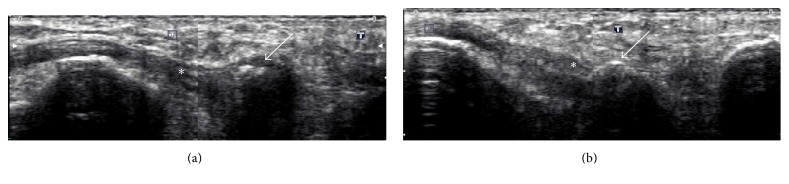
60-year-old female ultrasonography of the long axis of the peroneus longus tendon. (a) Right foot: complaint side demonstrating a thickened and heterogeneous peroneus longus tendon (asterisk) and irregular and fragmented os peroneum, associated with swelling of the surrounding soft tissues. (b) Left foot: contralateral side for comparison, demonstrating a preserved echotexture of the peroneus longus tendon (asterisk) and regular contours of the os peroneum without changes in the surrounding soft tissues.

We also performed comparative ultrasonography of the feet (Figure 3). It also identified irregularity and discontinuity of the right os peroneum (Figure 3(a)) and regular shape of this ossicle in the left (Figure 3(b)). We were also able to identify edema of the adjacent soft tissues and tendinopathy of the peroneus longus only on the right side (Figure 3(a)). We emphasize that the right os peroneum region coincided with the exact painful area.

The patient follows conservative treatment with medication and physical therapy. However, she reports only partial improvement of symptoms and most recently began a slightly painful condition on the left foot. It was not symptomatic when the imaging studies presented in this report were performed.

## 3. Discussion

There are different sesamoids and accessory ossicles in the skeleton. Some of them are known to be associated with painful syndromes, such as os trigonum, os navicular, and fabela. These syndromes may be caused by different etiologies such as trauma, infection, impact, and degenerative changes [3].

The os peroneum is an accessory ossicle, round or oval, within the substance of the peroneal tendon [1], and can be classified accordingly to Nwawka et al. and Blitz and Nemes as a sesamoid [2, 4]. Its histological structure is composed of different degrees of ossification and fibrous tissue [5].

The peroneus longus tendon is located proximal and posteriorly to the lateral malleolus on the lateral surface of the calcaneus, cuboid (along the midfoot), and distally inserting at the base of the first metatarsal and medial cuneiform [1, 6].

There are several causes for pain in the lateral aspect of the foot, including dislocation or subluxation of the peroneal tendon, injury, to the talofibular ligament or calcaneofibular ligament, or fractures in the fifth metatarsal, anterior process of the calcaneus, or cuboid [1].

The os peroneum fracture may be complicated by rupture of the peroneus longus tendon. The most common mechanism occurs with a strong contraction of the peroneus longus muscle in response to a sudden inversion or supination. Such contraction can compress the os peroneum against the cuboid, resulting in fracture and rupture of the peroneus longus tendon. It has been suggested that the presence of this ossicle can predispose to its distal rupture due to potential increased friction with adjacent structures [7]. Physical examination can reveal swelling over the cuboid, with pain in this area during palpation. The pain is usually exacerbated by plantar flexion and heel elevation stage during gait [7].

POPS has two main forms: acute and chronic. The acute form occurs as a result of trauma, commonly with ankle sprain or supination movement, resulting in fracture or diastasis of the os peroneum, which may or may not be associated with peroneus longus tendon rupture. Chronic presentation is closely linked to a healing process of a fracture with subsequent calcification, remodeling, or chronic diastasis of the os peroneum with a variable frequency of tenosynovitis of the peroneus longus tendon.

With MRI, the ossicle is usually isointense to bone marrow and presents with increased intrasubstantial signal within the peroneus longus tendon, typically close to the cuboid. Under ultrasonography, its identification is easily appreciated because of its typical bone appearance, as a curved echogenic focus with posterior acoustic shadow [8].

On radiographs, it is better identified in an oblique view of the foot. Both radiography and computed tomography may demonstrate displacement of the os peroneum from its usual position, fracture, or diastasis of a bipartite sesamoid. The displacement of the os peroneum is an indirect sign of a peroneal tendon rupture [2].

The radiographic differentiation between a fractured or split os peroneum may be difficult. In an acute event, fracture margins seem relatively nonsclerotic and bone fragments generally fit together, as “pieces of a puzzle.” In the bipartite sesamoid, margins become rounded and sclerotic. It is possible that over time due to remodeling, the edges of the fracture resemble the appearance of a split os peroneum. Brigido et al. suggested that a diastasis between fragments of os peroneum, greater than five millimeters, must indicate the diagnosis of fracture. US and MRI can also be used, especially to evaluate other possible associated abnormalities.

In the same study by Brigido et al., all bone fragments identified with US were hyperechogenic [7]. The evaluation of sesamoid fractures with MRI is difficult because of their small size and low signal. Bone marrow swelling can also complicate evaluation of fractures due to abnormal marrow signal intensity.

Therefore, early diagnosis and correct characterization of POPS are essential for an adequate management of these patients. Knowledge of its presentation through different imaging methods is very important during training of specialists in Radiology and Diagnostic Imaging.
